# Genetic risk-factors for anxiety in healthy individuals: polymorphisms in genes important for the HPA axis

**DOI:** 10.1186/s12881-020-01123-w

**Published:** 2020-09-21

**Authors:** Heléne Lindholm, India Morrison, Alexandra Krettek, Dan Malm, Giovanni Novembre, Linda Handlin

**Affiliations:** 1 Department of Biomedicine, School of Health Sciences, University of Skövde, Box 408, 54128 Skövde, Sweden; 2 Center for Social and Affective Neuroscience, Linköping University, Linköping, Sweden; 3 Department of Public Health, School of Health Sciences, University of Skövde, Skövde, Sweden; 4 Department of Internal Medicine and Clinical Nutrition, Institute of Medicine, Sahlgrenska Academy at University of Gothenburg, Gothenburg, Sweden; 5 Department of Community Medicine, Faculty of Health Sciences, UiT The Arctic University of Norway, Tromsø, Norway; 6 Department of Nursing Sciences, School of Health and Welfare, Jönköping University, Jönköping, Sweden

**Keywords:** Anxiety, Stress, HPA axis, Polymorphism, STAI

## Abstract

**Background:**

Two important aspects for the development of anxiety disorders are genetic predisposition and alterations in the hypothalamic-pituitary-adrenal (HPA) axis. In order to identify genetic risk-factors for anxiety, the aim of this exploratory study was to investigate possible relationships between genetic polymorphisms in genes important for the regulation and activity of the HPA axis and self-assessed anxiety in healthy individuals.

**Methods:**

DNA from 72 healthy participants, 37 women and 35 men, were included in the analyses. Their DNA was extracted and analysed for the following Single Nucleotide Polymorphisms (SNP)s: rs41423247 in the *NR3C1* gene, rs1360780 in the *FKBP5* gene, rs53576 in the *OXTR* gene, 5-HTTLPR in *SLC6A4* gene and rs6295 in the *HTR1A* gene. Self-assessed anxiety was measured by the State and Trait Anxiety Inventory (STAI) questionnaire.

**Results:**

Self-assessed measure of both STAI-S and STAI-T were significantly higher in female than in male participants (*p* = 0.030 and *p* = 0.036, respectively). For SNP rs41423247 in the *NR3C1* gene, there was a significant difference in females in the score for STAI-S, where carriers of the G allele had higher scores compared to the females that were homozygous for the C allele (*p* < 0.01). For the SNP rs53576 in the *OXTR* gene, there was a significant difference in males, where carriers of the A allele had higher scores in STAI-T compared to the males that were homozygous for the G allele (*p* < 0.01).

**Conclusion:**

This study shows that SNP rs41423247 in the *NR3C1* gene and SNP rs53576 in the *OXTR* gene are associated with self-assessed anxiety in healthy individuals in a gender-specific manner. This suggests that these SNP candidates are possible genetic risk-factors for anxiety.

## Background

Anxiety disorders are complex disorders defined by excess worry, hyperarousal, and fear that are counterproductive and debilitating [1]. Such disorders globally associate with socio-economic disadvantages, high demands at work, relationship difficulties, trauma and conflict [2], indicating that stressful events are major contributors to the development of anxiety disorders. The biological bases of these disorders depend partly on alterations in the hypothalamic-pituitary-adrenal axis (HPA axis). The HPA axis coordinates bodily reactions to stressful events and restores homeostasis. In response to stress, a hormonal cascade is triggered in the hypothalamus, where the release of corticotrophin-releasing hormone (CRH) stimulates the secretion of adrenocorticotropic hormone (ACTH) from the pituitary. Further in the signaling pathway to peripheral organs, ACTH then induces glucocorticoid (GC) synthesis in the adrenal glands [3]. Released GCs bind to intracellular glucocorticoid receptors (GRs), which after ligand binding translocate to the nucleus and thereby modify the expression of genes to help maintain homeostasis [4].

The activity of the HPA axis is regulated both through negative feedback from its own hormones and through neurotransmitters such as serotonin and oxytocin [5]. Serotonin is released from serotonin neurons ascending from the raphe nucleus in the midbrain to the paraventricular nucleus (PVN) in the hypothalamus [6], where it increases HPA axis activity. In contrast oxytocin, which is released from the posterior pituitary, decreases activity in the HPA axis and thereby acts as a buffering neuropeptide on the stress response. This helps the individual to restore homeostasis. Oxytocin also affects serotonin release in the amygdala by activation of oxytocin receptors expressed in serotonergic neurons [7, 8].

Genetic predisposition is another important aspect in the development of anxiety disorders, for which a heritability of approximately 30 to 50% has been reported [9]. Accordingly, many genes are associated with stress-related illnesses and anxiety. Genes related to HPA axis activity as well as its regulation are of special interest in this regard [10, 11]. For example, the *NR3C1* gene encodes the glucocorticoid receptor (GR) and the single nucleotide polymorphism (SNP) BclI rs41423247 has been associated with an increased risk of major depressive disorder [12]. The *FKBP5* gene encodes the FK506 binding protein 51, which regulates GR sensitivity and SNP rs1360780 associates with less efficient negative feedback inhibition on the HPA axis [13, 14]. In addition, SNP rs53576 in the oxytocin receptor gene (*OXTR*) associates with depressive symptomatology [15, 16], stress-responsive activity [17] and increased anxiety [18]. Furthermore, two polymorphisms in the serotonergic system are often studied in association with anxiety disorders and depression. First, a polymorphism (5-HTTLPR) in the promoter region in the serotonin transporter *SLC6A4* gene results in either a long allele (l) or a short allele (s) where the short allele associates with increased levels of stress [19, 20] and a higher mental vulnerability to social stressors and chronic diseases [21]. Second, the SNP rs6295 in the transcriptional control region of the serotonin receptor *HTR1A* gene has been associated with severe depression and anxiety [22].

Anxiety disorders are considered the sixth largest contributor to loss of non-fatal health globally [23] and hence there is a need to identify risk-factors for anxiety disorders. To date, the relationship between an individual’s anxiety and his/her genetic predisposition has been poorly investigated in healthy individuals [24]. Studying this relationship in healthy individuals removes the confounding of psychiatric symptoms, making it possible to identify possible genetic risk-factors for anxiety. This is important in the search for knowledge about which individuals that may be more susceptible to developing anxiety, and later on possibly also depression, and provide important information of the mechanisms underlying anxiety. Therefore, the aim of this exploratory study was to investigate possible relationships between genetic polymorphisms in genes important for the regulation and activity of the HPA axis and self-assessed anxiety measured with the instrument State and Trait Anxiety Inventory (STAI) in healthy individuals.

## Methods

### Participants and study design

DNA and STAI-responses were collected from 72 healthy volunteering participants, 37 females and 35 males (self-assessed health, non-clinical). Participants were in the age range of 19–40 years old (mean age females = 24.6 years, SD = 4.6; mean age males = 26.8 years, SD = 6.0). The participants were recruited from the student population at Linköping University through an exploratory functional magnetic resonance imaging (fMRI) based study with the overall aim to investigate the neural, endocrine and genetic correlates of affective touch. The self –assessment of the participant’s health was done through questions asked to the participants. Although the results presented in this paper focus on the genetic aspects in connection with the STAI questionnaire, the inclusion and exclusion criteria were designed to fit the overall aim of the fMRI based study. The inclusion criteria were that couples should have been in a romantic relationship for more than 1 year and the female should be between 19 and 40 years. Exclusion criteria were current use of contraceptives with estrogen, pregnancy, as well as ongoing or recently completed hormone therapy. Since the fMRI based study also included endocrine measurements it only included participants that had been through puberty but had not entered menopause, therefore the selection of the 19–40 year old participants.

Samples for DNA extraction were collected from both female and male participants separately and all participants answered the STAI questionnaire one-on-one without their partner present.

The study was approved by the Swedish Ethical Review Authority through the regional Ethical Review Board in Linköping, Sweden (2015/88–31) and all participants gave written consent prior to participation.

### DNA sampling and extraction

For the female participants, DNA was extracted from venous blood collected in EDTA-tubes using the DNeasy Blood and Tissue Kit (Qiagen, Hilden, Germany). For the male participants, DNA was extracted from saliva collected in collection tubes Oragene DNA OG-500 using the Oragene PrepIT L2G (DNA Genotek Inc., Ontario, Canada). In the fMRI based study, through which the participants were recruited (described above), the female and male participants had different roles; neural and endocrine responses were investigated in the female participants while receiving touch from their male partners. Therefore, DNA was obtained from different sources for female and male participants.

### Polymorphism analysis

Each polymorphism was analyzed with the most suitable and effective method as determined in our laboratory for that particular polymorphism. Details follow below.

#### Serotonin transporter *SLC6A4*, 5-HTTLPR

The DNA-fragment in the promoter region of the *SLC6A4* was amplified by polymerase chain reaction (PCR) using the following reaction mix: 40 ng of genomic DNA, 0.5 mM of each primer as previously described [21] (Table 1) and 10 μl GoTaq (Promega, Madison, Wisconsin, USA) in a reaction volume of 20 μl. After an initial denaturation step for 5 min at 95 °C, 35 cycles of denaturation at 95 °C for 30 s, annealing at 59 °C for 40 s and extension of 72 °C for 50 s were performed, followed by a final extension step of 72 °C for 5 min. PCR-products were separated on a 3.5% agarose gel to distinguish between the long allele (l), 440 bp, and short allele (s), 396 bp.

**Table 1:** Primers and probes used for the analyses of genetic polymorphisms in PCR and ddPCR

Gene	SNP	Primer Sequence 5′-3′/ ddPCR assay sequence (SNP in brackets)	Product (bp)	Polymorphism
*SLC6A4*	5-HTTLPR	F-ATGTCCCTACTGCAGCCTCC	s(396)/l(440)	(s)/(l)
		R-AGTCCGCGCGGGATTC		
*HTR1A*	rs6295	F-GGCTGGACTGTTAGATGATAACG	C(163) G(17 & 146)	C/G
		R-GGAAGAAGACCGAGTGTGTCAT		
*NR3C1*	rs41423247	CATTTGAACGTAAAATTTTGTTTTGCACCATGTTGACACCAATTCCTCTCTTAAAGAGATT		C/G
		**[C/G]**ATCAGCAGACATAACTTGTCTACTTTATGGCAAGAACCCTGTGAGCAAGACCTGTGTCTAA		
*FKBP5*	rs1360780	F-CTGCAAGTCCCCAAAATTTGAC		C/T
		R-ATCTCTTGTGCCAGCAGTAG		
*OXTR*	rs53576	GTCCCCCACACCTCGGGCACAGCATTCATGGAAAGGAAAGGTGTACGGGACATGCCCGAGG**[A/G]**TCCTCAGTCCCACAGAAACAGGGAGGGGCTGGGAAGCTCATTCTACAGATGGGGAAACAGT		A/G

Expected product size in base pairs depending on the polymorphism

*SNP* Single nucleotide polymorphism, *F* Forward primer, *R* Reverse primer, *bp* Base pairs

#### Serotonin receptor gene *HTR1A*, rs6295

A 163-bp fragment containing the SNP at nucleotide position HTR1A-1019 was amplified using the same setup of PCR-reaction and program as for *SLC6A4*. The reverse primer (Table 1) was designed to introduce a variable restriction site depending on if there is a C or a G in position HTR1A-1019, as previously described [22]. Subsequently, the introduced restriction site was detected by digesting 10 μl of the PCR product with the restriction enzyme BseGI in a total volume of 50 μl according to the manufacturer’s instructions (ThermoFisher, Waltham, Massachusetts, USA) and then separating the product on a 3.5% agarose gel. The undigested fragment (163 bp) carries the C and the digested one (146 bp/17 bp) carries the G in the same position.

#### Glucocorticoid receptor gene, *NR3C1*, (BclI) rs41423247

The gene variant of *NR3C1* SNP rs41423247 was analyzed with droplet digital PCR (ddPCR) mutation detection assay according to the manufacturer’s instructions (Bio-Rad, Hercules, California, USA). Probes were designed to discriminate between the alleles using two different fluorescent TaqMan probes (Table 1). The probe detecting the C allele was marked with the fluorophore HEX and the probe detecting the G allele was marked with the fluorophore FAM (Bio-Rad, Hercules, California, USA).

#### FK506-binding protein 51, *FKBP5*, rs1360780

The gene was amplified in the following PCR-reaction; 40 ng DNA, 0,2 mM dNTP, 0,4 mM of each primer (Table 1), 2 mM MgCl_2_ and 0,5 U of Taq-polymerase (New England Biolabs, Ipswich, Massachusetts, USA) in a total volume of 25 μl. Primers were designed using Primer-BLAST (https://www.ncbi.nlm.nih.gov/tools/primer-blast/). The same PCR program as for SLC6A4 was used. The SNP was then detected using Sanger sequencing.

#### Oxytocin receptor gene *OXTR*, rs53576

The gene variants of *OXTR* SNP rs53576 were analyzed with ddPCR mutation detection assay according to the manufacturer’s instructions (Bio-Rad, Hercules, California, USA). Probes were designed to discriminate between the alleles A and G using two different fluorescent TaqMan probes (Table 1). The probe detecting the A allele was marked with the fluorophore HEX and the probe detecting the G allele was marked with the fluorophore FAM (Bio-Rad, Hercules, California, USA).

### Anxiety measurement

The participants’ self-assessed anxiety was measured through STAI, a validated and frequently used questionnaire for measuring general anxiety [25]. There are two subscales in STAI; one determines state anxiety (S) which is a measure of how the person feels right now, the other measures the trait anxiety (T) which is the proneness for anxiety in the personality [25].

The STAI questionnaire is a self-administered test with 40 questions. State anxiety items include statements like “I am tense” and “I feel secure”. Trait anxiety items include “I worry too much over something that doesn’t really matter” and “I am a steady person”. Each item is scored on a 4-point Likert scale. The range 1 to 4 is from “not at all” to “very much so” for the STAI-S and from “almost never” to “almost always” for the STAI-T, with a total score range of 20 to 80. The median alpha reliability coefficients in healthy individuals for the STAI questionnaire (forms S and T) are 0.92 and 0.90, respectively [26, 27].

Missing data in the STAI-S and STAI-T questionnaires (maximum two missing values) was handled with hot deck imputation [28]. This was the case for two female participants, one in STAI-S (one item missing) and one in STAI-T (one item missing).

### Statistical analyses

All analyses were carried out using IBM SPSS 24 (SPSS Inc., Chicago, USA). We applied independent *t*-test to compare mean values of STAI-S and STAI-T between sexes and Chi-square test for calculating Hardy-Weinberg equilibrium for genetic polymorphisms. A probability level of < 0.05 was considered statistically significant both for testing gender differences in STAI and for Hardy-Weinberg equilibrium.Due to gender differences in the study participant’s STAI scores, the association analyses between STAI scores and genotypes in the candidate genes were carried out in female and male participants separately, using independent *t*-test. To control for multiple comparisons in the association analysis, a Bonferroni correction was performed, taking into account that five different SNPs were tested. Therefore results with a *p*-value < 0.01 were considered statistically significant for the association analysis.

## Results

### State and trait anxiety

Self-reported measure of STAI-S was significantly higher in female (*N* = 37, mean = 35.6, SD = ± 9.7) than in male (*N* = 35, mean = 31.6, SD = ± 5.7) participants *(*t = 2.2, df = 61.5, *p* = 0.030, two-tailed).

For STAI-T, we found significantly higher scores for females (*N* = 37, mean = 40.3, SD = ± 9.8) compared to males (*N* = 35, mean = 36.2, SD = ± 6.7) *(*t = 2.1, df = 67.5, *p* = 0.036, two-tailed). Therefore, female and male participants were considered separately in the following association analysis of the SNPs.

### Allele frequencies

Allele frequencies of the five polymorphisms in the whole cohort are shown in Table 2. Genotypes were distributed according to the Hardy-Weinberg Equilibrium for all SNPs except for 5-HTTLPR (*SLC6A4*).

**Table 2:** Allele and genotype frequencies of the five polymorphisms studied

Polymorphism	Allele	Allelle frequency	Genotype	Genotype frequency	Hardy-Weinberg Equilibrium
5-HTTLPR *N* = 72	s	0.451	s/s	0.264	χ2 = 4.26 *p* = 0.04
	l	0.549	s/l	0.375	
			l/l	0.361	
rs6295 *N* = 72	C	0.507	CC	0.261	χ2 = 0.01 *p* = 0.91
	G	0.493	CG	0.493	
			GG	0.246	
rs41423247 *N* = 72	C	0.364	CC	0.086	χ2 = 2.60, *p* = 0.11
	G	0.636	CG	0.557	
			GG	0.357	
rs1360780 *N* = 72	C	0.696	CC	0.507	χ2 = 0.83, *p* = 0.36
	T	0.304	CT	0.377	
			TT	0.116	
rs53576 *N* = 72	A	0.357	AA	0.072	χ2 = 2.32, *p* = 0.13
	G	0.643	AG	0.551	
			GG	0.373	

### Association analysis

**Fig. 1:**
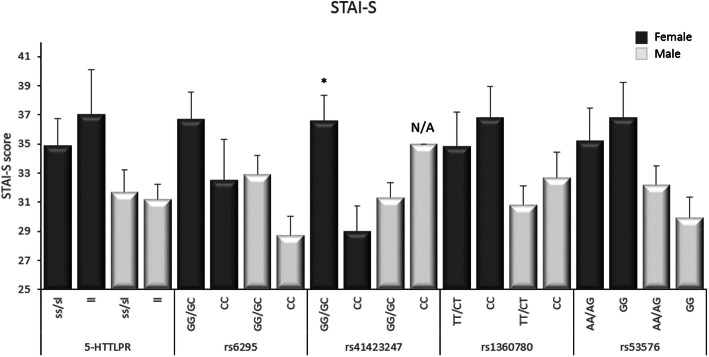
Associations between the different polymorphisms studied and mean scores (SEM) of STAI-S. * *p* < 0.01, N/A = only one participant

**Fig. 2:**
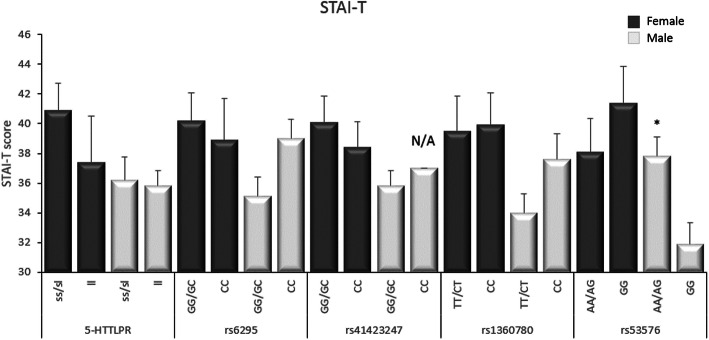
Associations between the different polymorphisms studied and mean scores (SEM) of STAI-T. * *p* < 0.01, N/A = only one participant

For the female participants there was a significant association between the score for STAI-S and SNP rs41423247 in the *NR3C1* gene. Carriers of the G allele had significantly higher scores in STAI-S compared to carriers of the C allele (t = − 3.077, df = 13.8, *p* = 0.008). No other significant associations between the investigated SNPs and the score of either STAI-S or STAI-T were detected for the female participants (Figs. 1 and 2).

For the male participants, there was a significant association between the score for STAI-T and SNP rs53576 in the *OXTR* gene. Carriers of the A allele had significantly higher scores in STAI-T compared to those that were homozygous for the G allele (t = 2.911, df = 28.6, *p* = 0.007). No other significant associations were detected between the SNPs in this study and the score of either STAI-S or STAI-T for the male participants (Figs. 1 and 2).

## Discussion

In this study, we investigated genetic risk-factors for anxiety by examining possible relationships between SNPs in genes important for the regulation and activity of the HPA axis and self-assessed anxiety measured with STAI in healthy individuals. We found significant gender-specific associations between STAI-S/T and SNPs in the *NR3C1* gene and the *OXTR* gene.

Female participants in our study scored significantly higher in both STAI-S and STAI-T compared to male participants. This was not unexpected, since females often exhibit higher scores in both STAI-S and T in self-reported assessments of anxiety, which may contribute to a higher incidence of anxiety disorders in women [29, 30]. Factors that contribute to such gender differences are different exposure to sex-hormones, particularly estrogens, and different social expectations and experiences [29]. Another aspect that might contribute to differences in STAI-scores between genders is that females may be more prone to admit anxiety than males, as doing so may be experienced as a threat to masculinity [31]. It is also possible that the preparations for the fMRI based study, through which our participants were recruited, for example invasive blood draws, had some effects on the anxiety levels of the females, especially for the STAI-S scores.

The association analyses showed a significant association in the female participants between STAI-S and the SNP rs41423247 in the *NR3C1* gene*,* which codes for the glucocorticoid receptor. Females with the CG/GG genotypes had significantly higher scores in STAI-S compared to females with the CC genotype. It has previously been shown that the G allele associates with increased sensitivity to glucocorticoids [32] and therefore an increased activity in the HPA axis, which could contribute to an increased risk of anxiety. In addition, another SNP on the same gene (rs6195) is associated with major depressive disorders in women, but not men [12]. These combined results strengthen the hypothesis that polymorphisms within the *NR3C1* gene can serve as genetic risk-factors for anxiety, at least state anxiety. Interestingly, these polymorphisms appear to be of special importance in females.

For the male participants, there was a significant association between high scores in STAI-T and the A allele in the SNP rs53576 of the *OXTR* gene. Males with at least one A allele had significantly higher scores in STAI-T compared to males with the GG genotype. This is in agreement with previous studies where the GG genotype associates with prosocial behavior [16] whereas the A allele is associated with anxiety and depression [15]. Therefore, SNP rs53576 in the *OXTR* gene emerges as a genetic risk-factor for trait anxiety which may be especially important for males.

In contrast, we found no association in neither males nor females between STAI-S/T and the SNP rs1360780 in the *FKBP5* gene, which codes for the FK506 binding protein 51 that regulates GR sensitivity. Ising et al. only found a relationship between *FKBP5* polymorphism and self-reported anxiety after a psychological stress test [14]. Since our study setup and aim did not include such a test, we could not establish any relationship between the polymorphism in the *FKBP5* gene and self-assessed anxiety after psychological stress. This indicates that polymorphisms in the *FKBP5* gene might be more important to study under stressful conditions and less important from a preventive perspective.

Furthermore, we did not find any association between STAI-S/T and the polymorphisms studied in the serotonergic system (5-HTTLPR in *SLC6A4* and rs6295 in *HTR1A*). Previous reports have also failed to determine an association between the polymorphisms in *SLC6A4* regarding depressive symptoms among humans with high social support (social compensation) [33, 34]. In our study, the inclusion criterion of “being in a romantic relationship” might have excluded individuals with low social support. The high social support among our participants may thus have influenced their self-reported anxiety, which might explain why we could not determine an association with the different genotypes in the serotonergic system.

In summary, our results point towards a gender differences in the polymorphisms in the genes controlling either the activity (*NR3C1* gene*)* or the regulation (*OXTR* gene) of the HPA axis. Our female participants clearly scored significantly higher in the STAI questionnaire than did their males counterparts. Previous studies have also demonstrated gender differences in the HPA axis [35] and that these differences can play a role in differentially forming the personality traits of females and males [36]. The reason for these gender differences are not yet fully understood, but the effects of sex-hormones on neurological development and function of different endocrine systems in humans may play a role [29]. Interestingly, estrogen has been identified as one important factor for gender differences in anxiety, since it has modulating effects on the HPA axis. However, the effects vary according to studies and preclinical and clinical populations [37]. In our study, we controlled for use of contraceptives with estrogen, pregnancy, as well as ongoing or recently completed hormone therapy. Therefore, any gender differences that might be due to differences in estrogen levels are influenced by endogenous estrogen levels only. In addition, both female and male participants were within the same age range which makes differences between genders not attributed to age differences. Our results highlight the importance of investigating female and male participants separately in these types of studies.

The validity of genetic association studies depends on the genotypes being in Hardy-Weinberg equilibrium. Violations of the Hardy-Weinberg equilibrium might signal errors in the analyzed data and problems in the interpretations of the genetic association data [38]. One limitation of our study is the limited number of participants, but since all genotypes except one were in Hardy-Weinberg equilibrium our study sample is likely to be representative and applicable to the population.

A novelty with our study is the preventive approach including only healthy, non-clinical participants. This means that the results presented here are not confounded by psychiatric health problems and can therefore help in identifying possible genetic risk-factors for anxiety. To further investigate the importance of the genotypes identified in our study, it would be interesting to proceed with future case-control studies with patients that suffer from anxiety.

## Conclusion

This study shows that SNP rs41423247 in the *NR3C1* gene and SNP rs53576 in the *OXTR* gene are associated with self-assessed anxiety in healthy individuals in a gender-specific manner. This makes these polymorphisms candidates for genetic risk-factors for anxiety.

## Data Availability

The datasets generated and/or analyzed during the current study are not publicly available due to risk of compromising individual privacy. The application and the written consent forms approved by the Swedish Ethical Review Authority through the regional Ethical Review Board in Linköping, Sweden, states that the data will only be available to the researchers within the project.

## Abbreviations

HPA axis: Hypothalamic-pituitary-adrenal axis
SNP: Single nucleotide polymorphisms
CRH: Corticotropin-releasing hormone
ACTH: Adrenocorticotropic hormone
GC: Glucocorticoid
GR: Glucocorticoid receptor
STAI: State and Trait Anxiety Inventory
PCR: Polymerase chain reaction
ddPCR: Digital droplet polymerase chain reaction
